# Nanoemulsion for improving solubility and permeability of *Vitex agnus-castus* extract: formulation and *in vitro* evaluation using PAMPA and Caco-2 approaches

**DOI:** 10.1080/10717544.2016.1256002

**Published:** 2017-02-06

**Authors:** Vieri Piazzini, Elena Monteforte, Cristina Luceri, Elisabetta Bigagli, Anna Rita Bilia, Maria Camilla Bergonzi

**Affiliations:** 1 Department of Chemistry, University of Florence, Sesto Fiorentino, FI, Italy and; 2 NEUROFARBA, Department of Neurosciences, Psychology, Drug Research and Child Health, Section of Pharmacology and Toxicology, University of Florence, Florence, Italy

**Keywords:** Nanoemulsion, *Vitex agnus-castus* L., solubility, PAMPA, Caco-2

## Abstract

The purpose of this study was to develop new formulation for an improved oral delivery of *Vitex agnus-castus* (VAC) extract. After the optimization and validation of analytical method for quali-quantitative characterization of extract, nanoemulsion (NE) was selected as lipid-based nanocarrier. The composition of extract-loaded NE resulted in triacetin as oil phase, labrasol as surfactant, cremophor EL as co-surfactant and water. NE contains until 60 mg/mL of extract. It was characterized by DLS and TEM analyses and its droplets appear dark with an average diameter of 11.82 ± 0.125 nm and a polydispersity index (PdI) of 0.117 ± 0.019. The aqueous solubility of the extract was improved about 10 times: the extract is completely soluble in the NE at the concentration of 60 mg/mL, while its solubility in water results less than 6 mg. The passive intestinal permeation was tested by using parallel artificial membrane permeation assay (PAMPA) and the permeation across Caco-2 cells after preliminary cytotoxicity studies were also evaluated. NE shows a good solubilizing effect of the constituents of the extract, compared with aqueous solution. The total amount of constituents permeated from NE to acceptor compartment is greater than that permeated from saturated aqueous solution. Caco-2 test confirmed PAMPA results and they revealed that NE was successful in increasing the permeation of VAC extract. This formulation could improve oral bioavailability of extract due to enhanced solubility and permeability of phytocomplex.

## Introduction

In the last years, the use of herbal medicines has been increased due to their therapeutic effects and fewer adverse effects as compared with the modern medicines. However, many herbal drugs and herbal extracts have low *in-vivo* activity due to their poor solubility, absorption and hence poor bioavailability, low stability or high metabolism.

Drug delivery systems represent good tools to formulate herbal drugs and to enhance their bioavailability. The novel formulations have remarkable advantages over conventional formulations of natural compounds and extracts, which include increase of solubility, bioavailability, stability, enhancement of intracellular uptake, modification of pharmacokinetics and bio-distribution, sustained delivery. The strategy of drug delivery systems has been applied not only to many synthetic drugs but also to phytopharmaceuticals, including both isolated compounds and purified extracts (Porter et al., 2008; Sermkaew et al., 2013; Bergonzi et al., 2013,2014; Righeschi et al., 2014; Esfanjani & Jafari, 2016; Isacchi et al., 2016).

The purpose of this study was to develop oral innovative formulation of VAC extract to improve the solubility of constituents and enhance its therapeutic efficacy.

In recent years, much attention has been focused on lipid-based nanovectors, namely solid lipid nanoparticles (SLN), nanostructured lipid carriers (NLC), self-emulsifying drug delivery systems (SEDDSs) and nanoemulsions (NEs) (Ibrahim et al., 2015). Lipid carriers promote absorption through GI tract, by ameliorating the solubility of drug and by increasing the solubilization capacity of the GI fluids (Porter et al., 2007,2008). In fact, lipid formulations avoid the dissolution step in comparison with an oral solid or suspension dosage form and provide significant improvement of oral absorption.

NEs are highly dispersed, stable, transparent formulations and easy to prepare. Furthermore, their nanoscopic dimensions allow a better absorption by the cell membranes. Finally, NEs showed the ability to solve the problems of solubility and stability of many synthetic drugs, phytotherapeutic, nutraceuticals and food additive (Čilek et al., 2006; Hu et al., 2011; Sermkaew et al., 2013; Bergonzi et al., 2014; Akhtar et al., 2016). These characteristics give them transparency and allow to formulate the drug as both aqueous solutions and as non-aqueous concentrates, diluted with water immediately before administration, or administered as such.

The main aim of this research was to formulate an o/w NE of VAC for oral administration, using food acceptable components, to increase solubility, stability and ameliorate intestinal permeability of extract’s constituents.

VAC is a native species of the Mediterranean region. It has a long tradition as an herbal remedy and it was used in ancient times not only as an aphrodisiac but also against diverse disturbances of the female genital system. Different classes of analytes, like essential oil, iridoids, flavonoids, phenolic acids and diterpenoids, have been reported from VAC fruits and preparations thereof (Hoberg et al., 1999; Proestos et al., 2006; Högner et al., 2013).

Therapeutic indications are premenstrual syndrome including symptoms such as mastodynia or mastalgia and menstrual cycle disorders (Berger et al., 2000; EMA, 2010). *In vitro* and *in vivo* experiments have demonstrated the dopaminergic and prolactin-inhibiting activities of VAC extract. Indeed, an increase in serum levels of prolactin is a frequent cause of menstrual irregularities, pre-menstrual syndrome and mastodynia (Jarry et al., 1994; Zahid et al., 2016).

The main characteristic constituents are: bicyclic diterpenes, iridoid glycosides (agnuside), lipophilic flavonoids (casticin, penduletin), hydrophilic flavones (luteolin, isovitexin) (Zahid et al., 2016).

The study also includes the optimization of HPLC–DAD–ESI/MS analytical method for qualitative and quantitative characterization of commercial VAC extract and the preparation of NE as lipid carrier of phytocomplex. Physical and chemical stabilities of optimized formulation were assessed during three months.

Finally, *in vitro* permeation or transport studies were performed by using different models such as parallel artificial membrane permeability assay (PAMPA) and Caco-2 cells line, after preliminary cytotoxicity studies. These *in vitro* results could provide useful information about dissolution and permeation/absorption aspects, which further could be correlated to *in vivo* behavior.

## Materials and method

### Materials

VAC dry extract BNO 1095 (DER 7-11:1), was supplied by Bionorica SE, Neumarkt, Germany. Agnuside, isovitexin and casticin HPLC grade, luteolin, apigenin, isoorientin, ac. 3,4-dihydroxybenzoic and ac. p-OH-benzoic were from Extrasynthese (Genay Cedex, France).

Olive oil was from Olivia (Badia a Settimo, Florence, Italy); wheat germ oil (*Triticum aestivum*) was purchased from Aboca (Sansepolcro, Arezzo, Italy); sunflower oil (*Helianthus annus*) was from Coop (Massarosa, Lucca, Italy); tween® 20, tween® 40, cremophor EL, dl-α-Tocopherol acetate (Vitamin E), triacetin, PEG 400, were purchased from Sigma-Aldrich (Milan, Italy); hempseed oil, almond oil, borage oil, glycerol, propylene glycol were from Galeno SrL (Comeana, Prato, Italy); oleic acid was from Farmitalia, Carlo Erba SpA (Milan, Italy); labrafil, labrasol ECH, capryol 90, transcutol HP were from Gattefossé (Saint Priest, France).

Ethanol analytical reagent CH_3_CN and MeOH HPLC grade and HCOOH (≥98%) were purchased from Sigma-Aldrich (Milan, Italy). Water was purified by a Milli-Q_plus_ system from Millipore (Milford, MA). Phosphotungstic acid (PTA) was from Electron Microscopy Sciences (Hatfield). Cholesterol, lecithin, dichloromethane, DMSO, 1,7-octadiene (≥98%), phosphate buffered saline (PBS) bioperformance certified, lipase from porcine pancreas, pepsin from porcine gastric mucose, bile salts, HCl were purchased from Sigma-Aldrich (Milan, Italy).

### Methods

#### Preparation of sample solution of VAC extract

Commercial extract (500 mg accurately weight) was suspended in 20 mL of methanol, ultrasonicated for 30 min and filtered (European Pharmacopoeia, 2014). The extraction was repeated three times. The filtrate was evaporated until dryness, and the residue was dissolved in methanol and analyzed by HPLC–DAD–MS analysis. Aqueous solubility was determined by dissolving until saturation commercial extract in deionized water at room temperature. The residue was eliminated by centrifugation and the solution was analyzed by chromatographic analysis.

#### HPLC/DAD/MS analyses

The quantitative analyses were carried out using a HP 1100 L liquid chromatograph equipped with a DAD detector and managed by a HP 9000 workstation (Agilent Technologies). A 150 mm × 4.6 mm i.d., 5 μm Zorbax Eclipse XDB, RP18 column was used. 20 μL of each sample were injected. Chromatography was carried out in gradient mode using a flow rate of 0.6 mL/min. The mobile phase was (A) formic acid/water pH 3.2 and (B) CH_3_CN. The multi-step linear solvent gradient used was: 0–5 min 15–20% B; 5–7 min 20–30% B; 7–10 min 30–40% B; 10–15 min 40–50% B; 15–20 min 50–80% B; 20–25 min 80–15% B; post time 10 min; oven temperature 30 °C, flux: 0.6 mL/min. The UV/Vis spectra were recorded in the range 200–700 nm and the chromatograms were acquired at 210, 260, 280, 350 nm.

For qualitative analysis, MS experiments were conducted using a LTQ equipped with an ESI interface (Finnigan LTQ, Thermofisher Scientific, Waltham, MA). Mass spectrometry and electrospray operating parameters were optimized for negative polarity. The following final settings were used: sheath gas flow rate (arb): 30, aux gas flow rate (arb): 5, sweep gas rate (arb): 5, capillary temp (°C): 290.00, capillary voltage (V): 16.93, tube lents (V): −99.72.

#### Method validation

HPLC–DAD method was validated according to the current international regulatory guidelines (ICH, 1996; EMA, 2012). In particular, linearity, accuracy, precision, reproducibility, repeatability of the method, limit of quantification (LOQ) and limit of detection (LOD) were assessed.

Calibration standard series of casticin, isovitexin and agnuside were analyzed in triplicate. Flavonoids of extract were expressed as isovitexin and casticin, agnuside was used to quantify iridoids. Calibration curves were obtained by plotting the peak areas versus the concentrations of each standard compound. The regression parameters (intercept, slope and correlation coefficient) were calculated by linear regression analysis.

The reproducibility of the injection integration procedure was determined for agnuside (1.96 mg/mL), casticin (1.97 mg/mL) and isovitexin (1.92 mg/mL). The solutions were injected ten times and the relative standard deviation (R.S.D. %) values were calculated.

Three solutions at different concentrations (2.08, 3.17 and 4.26 mg/mL of extract in methanol) were prepared in order to evaluate the repeatability of the method. Each solution was injected three times. The contents of iridoids and flavonoids derivatives were calculated in order to estimate the R.S.D. The same samples used to determinate the repeatability of the method were injected six times on three different days for the determination of intermediate precision.

Accuracy was assessed by analyzing the recovery percentage of standards into the three preparations of VAC extract. Three independent solutions of extract were prepared (2.00, 2.67 and 4.00 mg/mL) and each sample was injected three times. The average recovery percentage was calculated for each level of concentration. The accuracy was determined by spiking 100 μg/mL of casticin, isovitexin and agnuside separately to the three batches of the extract.

Specificity was defined as the non-interference by other analytes detected in the region of interest.

The peak purity was investigated by inspecting the UV-spectra and MS spectra at the beginning, at the apex and at the end of the peaks of each constituent of the extract. No deviations were reported.

The LOQ, defined as the lowest concentration of analyte that could be quantified with acceptable accuracy and precision, was estimated by injecting a series of increasingly dilute standard solutions until the signal-to-noise ratio was reduced to 10. The LOD corresponds to the concentration of analyte, which provides a signal equal to the background (blank) plus three times the standard deviation of the blank.

#### Solubility studies

The solubility of VAC extract in water, oils (olive oil, triacetin, wheat germ oil, sunflower oil, tocopherol acetate, hempseed oil, almond oil, borage oil, oleic acid), surfactants and co-surfactants (tween 20, tween 40, cremophor EL, labrafil, labrasol ECH, PEG 400, ethanol, glycerol, propylene glycol, capryol 90, transcutol HP) was determined by suspended an excess of extract in 5 mL of each vehicles. Each mixture was shaken at 25 °C for 24 h, and then was centrifugate at 13 148 × *g* for 10 min. After the removal of supernatant, the concentration of the components of the extract was determined by HPLC at 260 nm after dilution with methanol/dichloromethane (3:2).

#### Construction of ternary phase diagram

Pseudo-ternary phase diagrams were constructed using Chemix School vers. 3.60 software (Arne Standnes, Norway), in order to obtain the range of concentration in which NE exist. The pseudo-ternary phase diagrams were constructed using the water titration method.

Surfactant and co-surfactant were mixed at different weight ratios (*S*_mix_). For each *S*_mix_ ratio, pseudo-ternary phase diagram was elaborated by testing weight ratio of oil–*S*_mix_ of 0:100, 5:95, 10:90, 20:80, 30:70, 40:60, 50:50, 60:40, 70:30, 80:20 and 90:10. Each oil–*S*_mix_ mixture was diluted under vigorous stirring dropwise with water. After equilibrium, each sample was visually checked and determined as being clear NE, emulsion or gel.

Extract-loaded NE was prepared by dissolving powder into selected oil–*S*_mix_ mixture, adding the required quantity of water, and stirring to form a clear and transparent dispersion. The resulting NE was tightly sealed and stored at +4 °C temperature.

#### Solubility of VAC extract in NE

The NE capacity to solubilize VAC extract was investigated and compared with the solubility of extract in aqueous solution. In order to determine the maximum loading capacity of NE, increasing amounts of extract (ranging from 5 to 60 mg) were loaded to the optimized formulation. The mixture was stirred for 24 h at 25 °C under light shielding. The undissolved drug was removed by centrifugation at 13 148 × *g* for 10 min, then supernatant was taken and the constituents were quantified by HPLC–DAD at 260 nm after dilution with methanol/dichloromethane (3:2). The analyses were performed in triplicate.

#### Particle size analysis and Zeta--potential

Droplet size of the developed NE was measured by a dynamic light scattering (DLS), Ζsizer Nano series ZS90 (Malvern Instruments, Malvern, UK) equipped with a JDS Uniphase 22 mW He–Ne laser operating at 632.8 mm, an optical fiber-based detector, a digital LV/LSE-5003 correlator and a temperature controller (Julabo water-bath) set at 25 °C. Time correlation functions were analyzed to obtain the hydrodynamic diameter of the particles (Zh) and the particle size distribution (polydispersity index, PdI) using the ALV-60X0 software V.3.X provided by Malvern. Autocorrelation functions were analyzed by the Cumulants method, fitting a single exponential to the correlation function to obtain particle size distribution. Scattering was measured in an optical quality 4 mL borosilicate cell at a 90° angle, diluting the samples in distilled water. ζ potentials were measured using the same instrument; for all samples, an average of three measurements at stationary level was taken. The temperature was kept constant at 25 °C by a Haake temperature controller. ζ potential was calculated from the electrophoretic mobility, using the Henry correction to Smoluchowski’s equation.

#### Morphological characterization

Morphology and structure of NE were studied using transmission electron microscope (TEM, Jeol Jem 1010). 5 μL of NE was applied to a carbon film-covered copper grid after appropriate dilution. Most of the dispersion was blotted from the grid with filter paper to form a thin film specimen, which was stained with a phosphotungstic acid solution 1% w/v in sterile water. The samples were dried for 3 min and then were examined under a JEOL 1010 electron microscope and photographed at an accelerating voltage of 64 kV.

#### Stability studies

Empty and extract-loaded (60 mg/mL) samples were inserted into sealed glass vials and stored at 4 °C for 2 months, in order to evaluate the stability of NEs. Their chemical and physical stabilities were studied by monitoring the occurrence of phase separation, dispersed phase size and drug content at predetermined intervals by DLS and HPLC/DAD analyses.

Furthermore, in an effort to mimic physiological dilution process after oral administration, the NEs were diluted 10, 20 and 30-fold with distilled water (pH = 5.5). The dilutions were followed by gentle vortexing for 2 min at room temperature. The samples were analyzed by DLS to confirm the physical stability of the systems in terms of size and homogeneity. Chemical stability was assessed by quantifying total flavonoids and iridoids during 2 months.

NEs were also diluted up to 20-folds in media with different pH to mimic diverse environments met after oral administration.

The intragastric stability was tested in simulated gastric fluid (SGF) as described earlier (Aditya et al., 2013). Briefly, 5 mL of NE were suspended in 5 mL SGF (0.32% w/v pepsin, 2 g of sodium chloride and 7 mL HCl dissolved in 1 L water and pH adjusted to 1.8 using 1 M HCl) and incubated in a water bath at 37 °C under shaking speed of 100 strokes/min. After 2 h, sample was collected to analyze the size and PdI.

After digestion in simulated stomach condition, previous sample was subjected to digestion under simulated intestinal condition containing intestinal enzyme complex (lipase 0.4 mg/mL, bile salts 0.7 mg/mL, and pancreatin 0.5 mg/mL) and calcium chloride solution 750 mM at pH 7.0, 37 °C under shaking speed of 100 strokes/min. After 2 h digestion in simulated intestinal fluid (SIF), sample was collected and its physical stability was checked by DLS analysis.

#### *In vitro* parallel artificial membrane permeability assay

The assay is carried out in a 96-well, MultiScreen-IP PAMPA (Millipore corporation) filter plate. The ability of compounds to diffuse from a donor compartment, through a PVDF membrane filter pre-treated with a lipid-containing organic solvent, into an acceptor compartment is evaluated. 5 μL of lecithin (10 g/L) and cholesterol (8 g/L) in 1,7-octadiene solution were added to the filter of each well. Immediately after the application of the artificial membrane, 250 μL of drug containing donor solutions (saturated solution of extract in water and VAC loaded NE, 60 mg/mL) were added to each well of the donor plate. 250 μL of buffer (0.05 mL/mL DMSO/PBS, pH 7.4) were added to each well of the acceptor plate. Then, the acceptor plate was placed into the donor plate, ensuring that the underside of the membrane was in contact with buffer. The plate was covered and incubated at room temperature under shaking for 24 h and permeation was evaluated at 0.5, 2, 4 h.

#### Cell culture

The human colon carcinoma cell line Caco-2 was kindly provided by Prof. Masini (University of Florence). Cells were cultured in DMEM supplemented with 20% heat-inactivated fetal calf serum, 1% l-glutamine and 1% penicillin/streptomycin. Caco-2 cells were incubated at 37 °C in a humidified atmosphere containing 5% CO_2_. Upon reaching 80% confluence, cells were sub-cultured weekly at a split ratio of 1:3 by trypsinization.

#### MTS assay for cell viability

Viability analysis was performed using Cell Titer 96™ Aqueous One solution cell proliferation (3-(4,5-dimethylthiazol-2-yl)-5-(3-carboxymethoxyphenyl)-2-(4-sulfophenyl)-2H-tetrazolium) (MTS) assay kit (Promega Madison, WI). In brief, Caco-2 cells were transferred to flat bottom 96-well tissue culture plates (Corning) at a seeding density of 5 × 10^3^ cells/well and allowed to grow for 24 h under the conditions detailed above. For MTS assay, the culture medium was removed and replaced with fresh medium containing VAC loaded NE (60 mg/mL; 1:100–1:800 dilution) and the cells were incubated for further 4 or 24 h. Then the cells were exposed to MTS solution and allowed to incubate for 2 h at 37 °C. The product of the reaction was measured at 490 nm using a spectrophotometer (Multilabel Counter 1240 Victor 3, Perkin Elmer, Waltham, MA). Cell death was expressed as a percentage of values obtained from control, untreated cells calculated from three replicates of each NE dilution.

#### Cell culture for transport studies

For transport studies, cells were seeded at 50,000 cells/well in cell culture inserts with polyethylene terephthalate (PET) membranes (0.4 μm pore size, 1.12 cm^2^ growth surface area; BRAND, Italy). Culture medium (DMEM) was added to apical (AP) (0.5 mL) and basolateral (BL) (1.5 mL) side, and was replaced every other day for the first week and daily thereafter. Cells were let to differentiate for 18–21 days.

#### Monolayer integrity

The integrity of the layer was evaluated with the lucifer yellow (LY) permeability assay according to Iacomino et al. (2013). LY was diluted in transport buffer (HBSS with Ca^2+^, Mg^2+^, 25 mM HEPES, pH 7.4) and added to the AP compartment at a final concentration of 100 μM. After incubation at 37 °C for 1 h, the HBSS in the BL chamber was collected, and the concentration of LY was determined by using 485 nm excitation and 530 nm emission on a fluorescence plate reader (Multilabel Counter 1240 Victor 3, Perkin Elmer, Waltham, MA). The percentage of AP to BL permeability was calculated according to the following equation:

% permeability=Fluorescence in the BL-blankFluorescence LY-blank*100

The critical maximum flux of LY to identify leaky monolayers was estimated to be less than 3% of starting concentration.

#### Transport experiments

Transport study was performed according to Hubatsch et al. (2007). Before the transport study, the culture medium (DMEM) was replaced with preheated (37 °C) transport HBSS medium supplemented with 25 mM HEPES (pH 7.4). After the cell monolayer was equilibrated for 30 min at 37 °C, Caco-2 cells were treated for 1 h with different dilutions of VAC-loaded NE (1:5; 1:10; 1:15) in HBSS in the AP chamber, while the BL chamber contained only HBSS.

At predetermined time intervals (0–60 min), 0.3 mL of medium in the BL side was taken for HPLC analyses and replaced with the same volume of fresh HBSS. At the end of the experiments, the integrity of the layer was re-evaluated with the LY permeability assay as described above (Iacomino et al., 2013).

#### Statistical analysis

Experiments were repeated three times and results expressed as a mean ± standard deviation. Statistical significance was calculated by Student’s *t*-test with statistical significance level set at *p* < 0.05.

## Results

### *HPLC*–*DAD*–*ESI*–*MS analytical methods*

**Figure 1.**
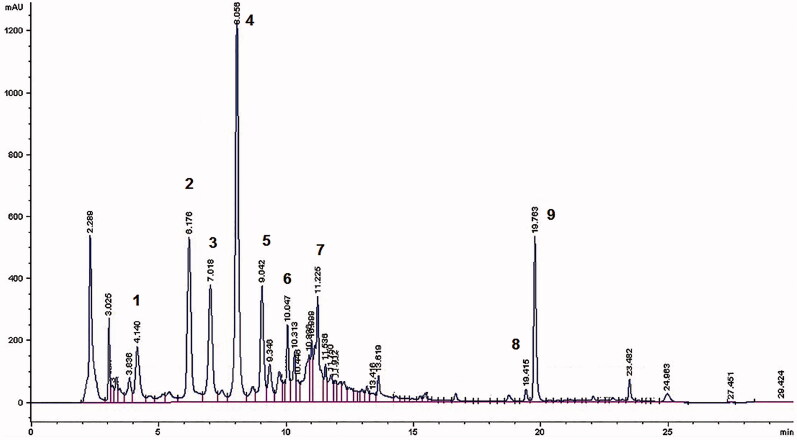
HPLC profile of VAC extract at 260 nm. 1. 3,4-Dihydroxybenzoic acid; 2. 4-Hydroxybenzoic acid; 3. Isoorientin; 4. Agnuside; 5. Isovitexin; 6. Luteolin; 7. Apigenin; 8. Penduletin; and 9. Casticin.

The first step of investigation was the HPLC–DAD–ESI–MS characterization of VAC extract. The wavelengths of 210, 260, 280 and 350 nm were selected for acquiring chromatograms of iridoid glycosides (agnuside), benzoic acid derivatives, flavonoids (isoorientin, isovitexin, luteolin, apigenin, penduletin, casticin), diterpenoids. Various linear gradients of acetonitrile–water and methanol–water were investigated at different flow-rates, by using various columns and temperature conditions, in order to achieve better chromatographic separation. Finally, the gradient programme described in the experimental part was selected, because all the peaks resulted clearly separated. Figure 1 reports the HPLC–DAD profile of the methanolic extract at the wavelength of 260 nm.

The identification of compounds was obtained by comparing UV spectrum, retention time and MS data with those of standards or with the data reported in the literature.

The HPLC–DAD method was validated according to the criteria established by ICH guidelines (1996), relatively to the following compounds: agnuside, isovitexin and casticin.

#### Linearity

Linearity range of response was determined for agnuside, isovitexin and casticin. They showed a linear response from 1 to 1000 μg/mL and all the curves had coefficients of linear correlation *R* ^2^ ≥ 0.999.

Additionally, the slope R.S.D. % values was lower than 1.5%, in particular for the casticin was 0.69%, for the agnuside was 0.24% and for isovitexin was 0.51% which indicated a high accuracy of the method.

#### Reproducibility

The reproducibility of the injection integration procedure was determined for agnuside (1.96 mg/mL), casticin (1.97 mg/mL) and isovitexin (1.92 mg/mL). The solutions were injected ten times and the relative standard deviation (R.S.D. %) values were calculated.

R.S.D. % of agnuside was 0.37%, the value for casticin was 0.58% and for isovitexin was 0.66%.

#### Repeatability of the method

Agnuside (R.S.D. % 0.74%, 1.60% and 0.83%, respectively), casticin (R.S.D. % 0.48%, 0.18% and 0.64%) and isovitexin (R.S.D. % 1.92%, 1.76% and 1.04%).

#### Intermediate precision

Results were reported in Table 1.

**Table 1.** Intermediate precision: RSD % values intraday and interday.

		RSD % Intraday			RSD % Interday		
Extract (mg/mL)	Day	Agnuside	Casticin	Isovitexin	Agnuside	Casticin	Isovitexin
2.08	1	1.672	1.807	0.704	1.632	0.919	0.878
	2	1.949	0.429	0.247			
	3	1.276	0.523	1.685			
3.17	1	1.835	0.211	1.984	1.776	1.281	2.348
	2	1.975	2.487	2.616			
	3	1.519	1.203	2.444			
4.26	1	1.365	0.863	0.364	1.574	0.749	1.362
	2	1.785	0.859	1.491			
	3	1.574	0.525	2.231			

#### Accuracy

The recovery percentages of standards spiked into VAC extract at three different concentrations were calculated. Three independent solutions of extract were prepared (2, 2.67 and 4 mg/mL) and each sample was injected three times. The average percentage recovery was calculated for each level of concentration. The accuracy was determined by spiking 100 μg/mL of casticin and isovitexin separately to the three batches of the extract. The percentages recovery of casticin standard spiked into three preparations were 103.07 ± 0.43%; 94.01 ± 0.67% and 103.97 ± 0.82%, respectively; for isovitexin were 105.72 ± 0.36%; 114.70 ± 1.19% and 126.89 ± 1.96%, respectively and for agnuside resulted 102.04 ± 0.57%, 105.38 ± 0.67% and 113.62 ± 0.52%, respectively.

#### Specificity

The peak purity was investigated by inspecting the UV-spectra and MS spectra at the beginning, at the apex and at the end of the peaks of each constituent of the extract. No deviations were seen.

#### Detection (LOD) and quantitation limit (LOQ)

LOD and LOQ of agnuside, casticin and isovitexin were determined by calculation of the signal-to-noise ratio. LOD resulted 0.98 ng for agnuside, 0.98 ng for casticin and 3.84 ng for isovitexin. LOQ resulted 1.96 ng for agnuside, 1.97 ng for casticin and 5.76 ng for isovitexin.

### Solubility study

To find out appropriate constituents of NE, the solubility of extract was determined in various oils, surfactants and co-surfactants. The excipients chosen needed to be pharmaceutically acceptable. Higher solubility of the substances in the oil phase was another important criterion, as it would help the NE to maintain the product in solubilized form.

Selected oils were olive oil, triacetin, wheat germ oil, sunflower oil, tocopherol acetate, labrafil (oleoyl macrogol-6-glycerides), hempseed oil, almond oil, borage oil, oleic acid.

The surfactant was tween 20, tween 40, cremophor EL, labrasol ECH, PEG 400 and ethanol, glycerol, propylene glycol, capryol 90, transcutol HP were used as co-surfactant. The solubility was assessed by using the HPLC method as previously reported.

The solubility’s results were reported in Table 2 and expressed as total flavonoids and total iridoids.

**Table 2.** Solubility of VAC constituents in different vehicles (mean ± S.D., *n* = 3).

	Flavonoids (mg/mL)	Iridoids (mg/mL)
Oils		
Olive oil	0.107 ± 0.046	–
Triacetin	0.136 ± 0.036	–
Tocopheryl acetate	0.008 ± 0.001	–
Labrafil	0.014 ± 0.003	0.01
Sunflower oil	0.058 ± 0.001	–
Hempseed oil	0.057 ± 0.010	–
Almond oil	0.022 ± 0.006	–
Wheat germ oil	0.066 ± 0.017	–
Borage oil	0.053 ± 0.013	–
Oleic acid	0.082 ± 0.001	–
Surfactant/cosurfactant		
Tween 20	0.080 ± 0.016	0.065 ± 0.001
Tween 40	0.078 ± 0.016	0.023 ± 0.005
Cremophor EL	0.367 ± 0.008	0.303 ± 0.022
Labrasol	0.178 ± 0.012	0.130 ± 0.015
Capryol 90	0.057 ± 0.011	0.019 ± 0.002
PEG 400	0.214 ± 0.021	0.228 ± 0.041
Transcutol HP	0.206 ± 0.008	0.123 ± 0.037
Ethanol	0.163 ± 0.017	0.142 ± 0.017
Propylene glycol	0.170 ± 0.006	0.172 ± 0.062
Glycerol	0.043 ± 0.001	-

The aqueous solubility of flavonoids resulted 0.313 ± 0.01 mg/mL (isoorientin 0.090 ± 0.011 mg/mL, casticin 0.031 ± 0.010 mg/mL, apigenin 0.077 ± 0.009 mg/mL, luteolin 0.079 ± 0.017 mg/mL, isovitexin 0.036 ± 0.014 mg/mL) and iridoids 0.172 ± 0.02 mg/mL (agnuside).

Oil solubility of extract is low and only flavonoids can be quantified. Surfactants showed a higher solubilizing effect than oils in the case of both flavonoids and iridoids. This is probably because they are amphiphilic molecules with affinity for both polar groups and lipophilic groups of the molecules. The highest values were obtained with cremophor EL transcutol HP and PEG 400.

Selected media differently influences the solubility of the constituents, compared with water. In particular, olive oil, oleic acid, hemp oil, PEG 400, ethanol, propylene glycol and transcutol increased the solubility of casticin; transcutol, propylene glycol, PEG 400 and tween 40 showed their positive effect on solubility of apigenin and isovitexin, while PEG 400 and propylene glycol ameliorated the solubility of isoorientin. PEG 400 and chremophor increased solubility of agnuside. The solubility into single vehicles resulted lower than water, but a synergic effect of NE was observed for all types of constituents, both polar and lipophilic, as further reported.

The first NE considered after solubility results contained olive oil as lipophilic phase, ethanol, propylene glycol, cremophor EL or transcutol HP as co-surfactant, and tween 20 as surfactant, but no clear and transparent systems were obtained, also at high surfactant concentrations (efficiency of the surfactant >90% w/w and the water solubilization capacity <1). This is due to the fact that olive oil contains long-chain triglycerides of oleic acid and, in contrast to mineral oil, it is polar. However, molecular weight of both of oils is most probably too high to assist in the formation of a NE.

Then, triacetin was tested as oil phase, and labrasol and cremophor EL as surfactant and co-surfactant, respectively. The ratio of oil to *S*_mix_ was changed during preformulation study, in particular, *S*_mix_ 1:1, 1:2, 2:1, 1:2.5, 1:3, 1:4 were considered. The optimized NE was assessed for clarity and transparency by visual inspection.

### Pseudo-ternary phase diagram study

**Figure 2.**
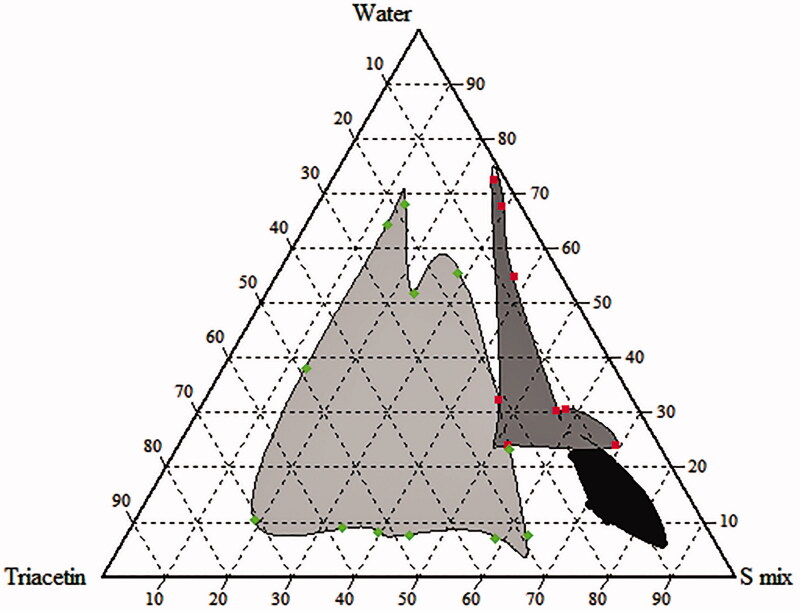
Pseudo ternary phase diagram of microemulsion (*S*_mix_: 1:1, Labrasol and Cremophor EL). Light gray: turbidity; gray: NE; and black: gel.

Phase diagram was constructed in order to obtain the concentration range of components necessary for the existence of NE. The pseudo-ternary phase diagram was built using water titration method. After equilibrium, each sample was visually checked to define if clear NE, emulsion or gel were present. Figure 2 shows the pseudo-ternary diagram with a field of existence of NE. The selected composition (1:2 oil–*S*_mix_ and *S*_mix_ 1:1) was: triacetin (20%) as oil phase, labrasol (22%) as surfactant, cremophor EL (22%) as co-surfactant (*S*_mix_), respectively and water (36%). The NE contains 60 mg/mL of extract. Distilled water was used as an aqueous phase and it was added drop by drop, under gentle agitation, to each oily mixture.

### Characterization of extract loaded NE

A lower diameter of the drops of oil (<100 nm) (Tenjarla, 1999) and the transparency of the system confirm the presence of NE. DLS evidences a homogeneous system, with a narrow size distribution for all samples, low PdI and mean diameter values. Empty NE shows an average diameter of 11.50 ± 0.16 nm and PdI 0.152 ± 0.023; extract loaded NE has sizes of 11.82 ± 0.125 and PdI 0.117 ± 0.019. The dimensional data were compared with those obtained by TEM analysis, which confirmed the presence of dispersed droplets with size less 20 nm.

### Solubility of VAC extract into NE

The solubility of the extract into NE was determined by introducing increasing amounts of extract (from 5 to 60 mg) in the formulation (Table 3). The samples were analyzed by HPLC, after 48 h under magnetic stirring at RT. NE improved the solubility of the extract about 10 times, compared with that in water. The extract dissolves completely in the NE until 60 mg/mL, while its aqueous solubility results less than 6 mg. Table 3 reported the quantity of constituents solubilized into NE with increasing amount of added VAC extract. The solubility of both classes of constituents was enhanced about 10-folds.

**Table 3.** Solubility of VAC extract into NE.

Sample	Total flavonoids (mg/mL)	Total iridoids (mg/mL)
5 mg/mL of extract	0.26	0.11
10 mg/mL of extract	0.41	0.16
15 mg/mL of extract	0.75	0.26
40 mg/mL of extract	2.41	0.60
60 mg/mL of extract	3.47	1.75
Water	0.31	0.17

### Stability study

Empty and extract-loaded NEs were stored away from light at 4 °C for approximately 2 months, in order to determine their stability. The physical stability was measured by monitoring size of dispersed phase by using DLS; chemical stability was assessed by quantifying total flavonoids and iridoids. Furthermore, for each time point, macroscopic observations were performed to check the transparency of the sample and the occurrence of possible coalescence, creaming and separation phenomena.

Based on visual identification, empty or extract-loaded NE remained transparent for 2 months without the occurrence of phase separation at 4 °C. Furthermore, NEs can be diluted with aqueous solution without destroying their structure for 24 h. This aspect is important to define the solubilization capacity, because NE will be diluted by water in the gastrointestinal tract upon oral administration, with possible drug precipitation. Furthermore, it is a confirmation that we are dealing with a NE and not with micelles that would collapse with the dilution.

Empty NE showed a good physical stability; they remained transparent and the droplet size (11.50 ± 0.163) and PdI (0.145 ± 0.03) were unaffected. Select formulation was also stable at room temperature for 30 days, without showing phase separations or changes in the dimensions of dispersed phase.

NE containing VAC extract showed also a good physical stability for 60 days; they remained transparent, homogenous with constant droplet size (from 11.82 nm and PdI 0.117 to 11.20 nm and PdI 0.136).

**Figure 3.**
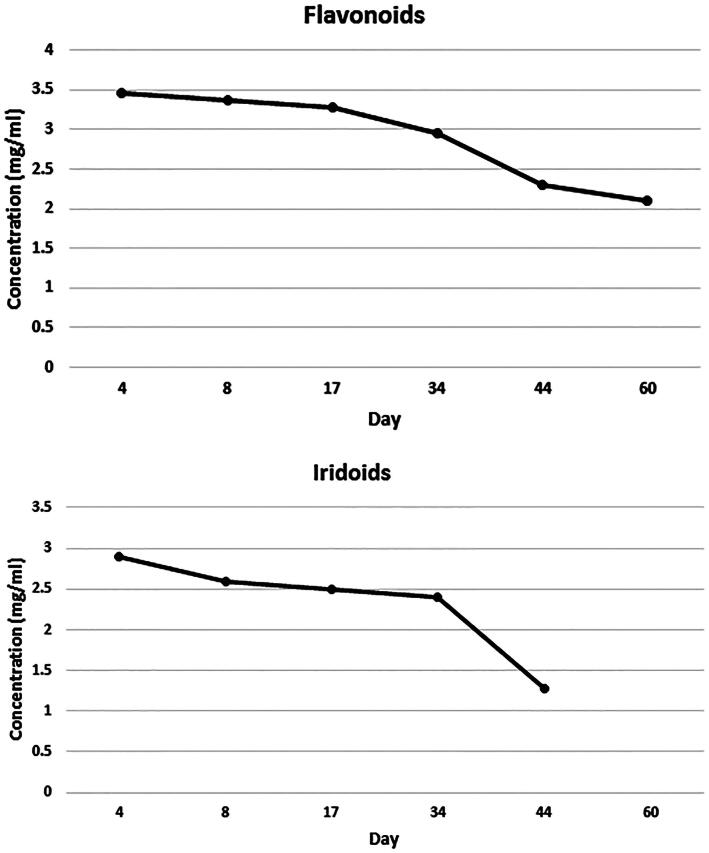
Chemical stability of flavonoids and iridoids of VAC extract formulated into NE after storage at 4 °C for 2 months (each data point represents the average of three samples).

Figure 3 evidenced chemical stability of flavonoids and iridoids. As evidenced from the results, the stability of formulation is limited to 34 days, after which there is a partial degradation of both classes of constituents.

Furthermore, in an effort to mimic physiological dilution process after oral administration, the NE used *in vitro* test was diluted up to 20-folds in simulated intestinal fluid and simulated gastric fluid. DLS analyses confirmed the physical stability of system. In particular, mean diameters and polydispersity values resulted unmodified at tested pHs in comparison with empty formulation (not diluted or diluted up to 20-folds in water, pH 5.5). Indeed, at pH 1.2 the sizes were 11.82 ± 0.13 nm and Pd 0.117 ± 0.019; at pH 5.5 the sizes were 11.75 ± 0.28 nm with Pd 0.218 ± 0.025, and at pH 6.8 the sizes resulted 11.99 ± 0.54 nm and Pd 0.208 ± 0.034. Values are the mean ± S.D. of three independent experiments.

### *In vitro* parallel artificial membrane permeability assay

**Figure 4.**
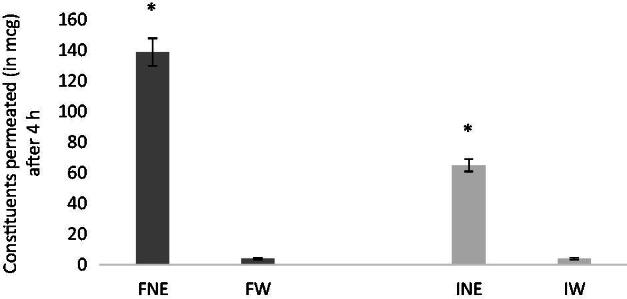
Comparative PAMPA permeability: micrograms of total flavonoids (F) and iridoids (I) of extract of VAC permeated from NE and from saturated aqueous solution (W). Average values ± S.D. of experiments carried out in triplicate are presented. *p < 0.05.

PAMPA assay is a method for predicting passive intestinal absorption (Kansy et al., 1998). PAMPA test is robust and reproducible and relatively fast (4–16 h) (Hiremath et al., 2009; Righeschi et al., 2016). In the last decades, it resulted as helpful complement and alternative to Caco-2 assay in the pharmaceutical research (Bermejo et al., 2004; Kerns et al., 2004; Sandhya et al., 2008). The experiment was carried out measuring the ability of VAC constituents to diffuse from donor compartment, containing NE formulation, to acceptor compartment, through a PVDF membrane. NE provided improved permeability of both flavonoids and iridoid agnuside after 4 h, in comparison with a saturated aqueous solution used as control (Figure 4). This fact is surely due to high solubilizing effect of NE on the constituents of extract, as previously reported in Table 3. The high amount of extract solubilized in the donor compartment promotes an increased passage through the membrane in the acceptor compartment.

The effective permeability of flavonoids and iridoids was calculated as reported in the literature (Chen et al., 2008; Hiremath et al., 2009). P_e_ was 46.5 ± 5.21 × 10^−6 ^cm/s for flavonoids and 41.29 ± 6.45 × 10^−6 ^cm/s for iridoid. In the case of aqueous solution, P_e_ was 14.05 ± 1.21 × 10^−6 ^cm/s for flavonoids and 29.49 ± 2.35 × 10^−6 ^cm/s for iridoids. The values confirmed that NE also increased the permeability of the extract compared with aqueous solution.

### Caco-2 permeability study

Monolayer of Caco-2 was used as a model to study the drug absorption across the intestinal epithelium. This well-differentiated human intestinal epithelial cell line is cultivated for studies of the transepithelial transport of drugs. In general, the aim is to investigate if a drug is actively or passively transported across the intestinal epithelium and, in the case of active mechanism, to identify the relevant carrier (Yee, 1997; Artursson et al., 2001).

Caco-2 cells are most widely and successfully used permeation model, that also allows to investigate paracellular permeability and active efflux (Balimane et al., 2006). The potential cytotoxicity of VAC-loaded NE on Caco-2 cells was tested in order to find the highest non/low toxic concentrations to be used in transport experiments. The percentage of cell death after exposure to vitex NE for 24 h at the final concentration of 1:600–1:800 was negligible compared with control (untreated cells). We also tested concentrations from 1:100 to1:300 for a short exposure time (for 4-h). With this approach, a low to moderate cytotoxicity ranging from 5 to 20% was observed. However, in order to overcome the limits of detection of HPLC analysis, the concentration of 1:100 was considered acceptable for transport experiments.

**Figure 5.**
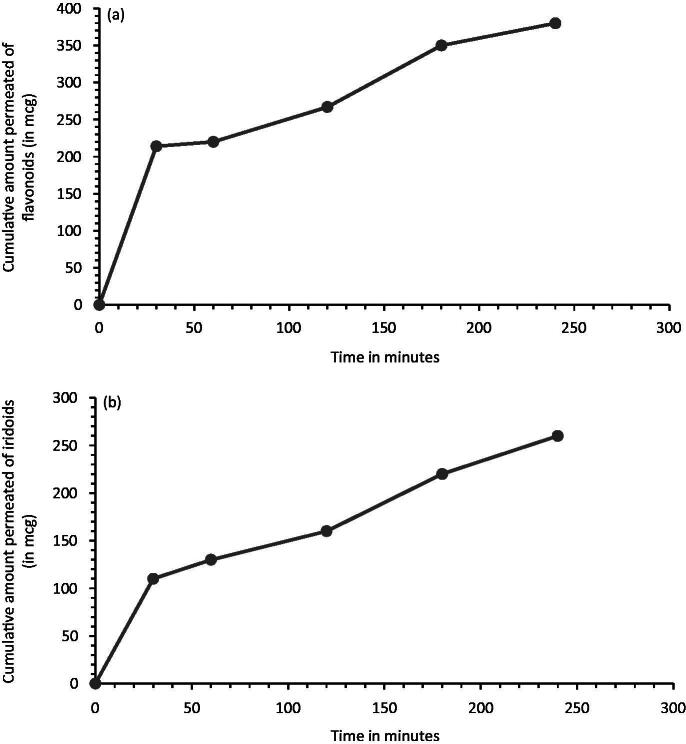
Caco-2 permeability of flavonoids (a) and iridoids (b) loaded into NE (each data point represents the average of three samples).

NE provided enhanced flavonoids and iridoids permeability in Caco-2 model, as evidenced in Figure 5. The apparent permeability values (P_app_) was 25.0 ± 3.4 × 10^−6 ^cm/s for flavonoids and 24.0 ± 1.8 × 10^−6 ^cm/s for iridoids. Thus, the flavonoids and iridoids in the NE exhibited the P_app_ values, necessary for complete intestinal absorption in humans (Artursson & Karlsson, 1991). Whereas, the VAC extract has not the permeability characteristics that could ensure this process. In the case of aqueous solution, only flavonoids (isovitexin and casticin) resulted detectable into acceptor compartment after 4 h (about 10 μg), with a P_app_ corresponding to 8.1 ± 0.9 × 10^−6 ^cm/s. Agnuside are not detectable after 2 h, while its P_app_ resulted 10.3 ± 2.1 × 10^−6 ^cm/s after 4 h. The recovery of flavonoids and iridoids was above 85% respectively, suggesting that the obtained P_app_ values were reliable. A recovery above 80% is required for an acceptable *in vitro* prediction of P_app_ values (Hubatsch et al., 2007).

There was a good correlation between PAMPA and Caco-2 permeability results. Both models showed that the permeation of extract resulted improved when it is formulated in NE. Good correlation between PAMPA and Caco-2 model indicated that the passive diffusion may be an important mechanism for permeation. As previously reported, PAMPA and Caco-2 permeation data have a good correlation to human oral absorption (Kerns et al., 2004). Given the characteristics of the two methods, these assays can be applied synergistically for efficient and rapid investigation of permeation mechanisms in preformulation studies, not only in the case of single molecules, but also for complex matrices, such as extracts and their formulations, as reported in this study.

## Conclusions

The VAC extract was formulated into NE. This formulation provides enhanced dissolution of the main constituents of the extract. The physical nature of these systems, mechanism of drug entrapment, as well as the physicochemical interactions of constituents determine their substances solubilization capacity and physical stability during storage and upon dilution.

The optimized formulation was obtained with triacetin as oil phase, labrasol and cremophor EL as surfactant and co-surfactant, respectively. It resulted an appropriate formulation in terms of size, polydispersity, encapsulation efficiency and its positive influence on the solubility of extract (60 mg/mL). NE can be diluted with aqueous buffer and it was stable for at least two months at 4 °C.

The *in vitro* transport studies, PAMPA and Caco-2 models, revealed that the optimized formulation was successful in enhancing the permeation both of flavonoids and iridoids, with respect an aqueous solution. The increase of *in vitro* permeation appears to be due to the effect on improved solubility/dissolution of extract.

After oral administration, the solubility and gastrointestinal permeability are fundamental parameters that control the rate and the extent of absorption and bioavailability. Consequently, *in vitro* dissolution has been recognized as an important stage in drug development; hence, the improvement of the dissolution rate of poorly soluble drug and of their bioavailability is an important challenge to pharmaceutical scientists. PAMPA and Caco-2 can synergistically provide invaluable information about permeability of single molecules and more complex substrates, such as extract and formulations. PAMPA assay is a particularly helpful complement to cellular permeability models, such as Caco-2, for its speed, low cost and versatility.

Thus, from the *in vitro* results, NE proved to be a promising oral delivery system for enhancing solubility and absorption of VAC extract.
